# Spontaneous migration of peripherally inserted central catheter into the azygos vein during postoperative gastrointestinal dysmotility: A case report

**DOI:** 10.1097/MD.0000000000033921

**Published:** 2023-06-02

**Authors:** Weinan Liu, Wei Wei, Yang Wang, Bing Liu, Yan Pang, Wenyan Sun

**Affiliations:** a Department of General Surgery, Peking Union Medical College Hospital, Chinese Academy of Medical Science & Peking Union Medical College, Beijing, China; b Department of Clinical Nutrition, Peking Union Medical College Hospital, Chinese Academy of Medical Science & Peking Union Medical College, Beijing, China.

**Keywords:** azygos vein, catheter dysfunction, catheter migration, peripherally inserted central catheter, postoperative gastrointestinal dysmotility

## Abstract

The spontaneous migration of the peripherally inserted central catheter (PICC) is the displacement of the PICC tip from a satisfactory documented position in the superior vena cava (SVC) into its adjacent veins after several days or months of PICC insertion, and most frequently occurs in the ipsilateral internal jugular vein. However, it is rarely reported to detect migration of PICC tip into the azygos vein in patients who suffered from gastrointestinal dysmotility after abdominal surgery. We report 2 cases of spontaneous malposition of PICC into the azygos vein here and discuss the predisposing factors and processing procedures of this condition.

**Patient concerns:**

Two female patients with pancreatic disease were inserted PICCs on the left limbs before the abdominal surgery. After the surgery, 1 patient suffered from gastroparesis, and the other suffered from constipation. The nurses found that blood could not be aspirated from the PICCs while normal saline could be injected through the PICCs smoothly.

**Diagnoses:**

We identified the position of the PICC tip step-by-step, using ultrasound, intracavitary electrocardiogram, and chest X-ray, and confirmed that the tip of the PICC migrated into the azygos vein.

**Interventions:**

The patients were placed in the semi-reclining position from the supine position, and blood could be easily aspirated from the PICC after flushing with the push-pause flush technique. Intracavitary electrocardiogram displayed the elevated P, indicating that the PICC tip reentered the SVC and was at the lower 1/3 of SVC.

**Outcomes:**

The PICCs of the 2 patients functioned well afterward and were removed after the parenteral nutrition support was completed.

**Lessons:**

It is critical to assess the function of the PICC before every time of infusion. For patients who undergo abdominal surgery with PICC on the left side, when they had gastrointestinal dysmotility combined with PICC dysfunction, the possibility of spontaneous migration of PICC tip into the azygos vein should be considered.

## 1. Introduction

The peripherally inserted central catheter (PICC) has been widely used in clinical practice to meet the needs for infusion of irritants/vesicants (such as chemotherapy and parenteral nutrition), invasive hemodynamic monitoring, or infusions in patients with poor condition of peripheral intravenous access.^[1]^ The tip of the PICC should be located in the lower third of the superior vena cava (SVC) at the Cavo atrial junction, and its location can be confirmed using posterior/anterior (PA) chest X-ray film and intracavitary electrocardiogram (ECG) technology.^[2]^ The spontaneous migration of the PICC is the displacement of the PICC tip from a satisfactory documented position in the SVC into its adjacent veins after several days or months of PICC insertion. It most frequently occurs in the ipsilateral internal jugular vein on the side of the catheter, and may also occur in the ipsilateral brachiocephalic vein, the ipsilateral subclavian vein, or the ipsilateral axillary vein.^[3]^ To identify the spontaneous PICC migration is of great significance because malposition of the PICC tip can induce infectious and thrombotic complications, even perforation of the vessel if not found and treated in time.^[4,5]^ Here, we report 2 cases of spontaneous malposition of PICC into the azygos vein and discuss the predisposing factors and processing procedures of this condition.

## 2. Case report

**Figure 1.**
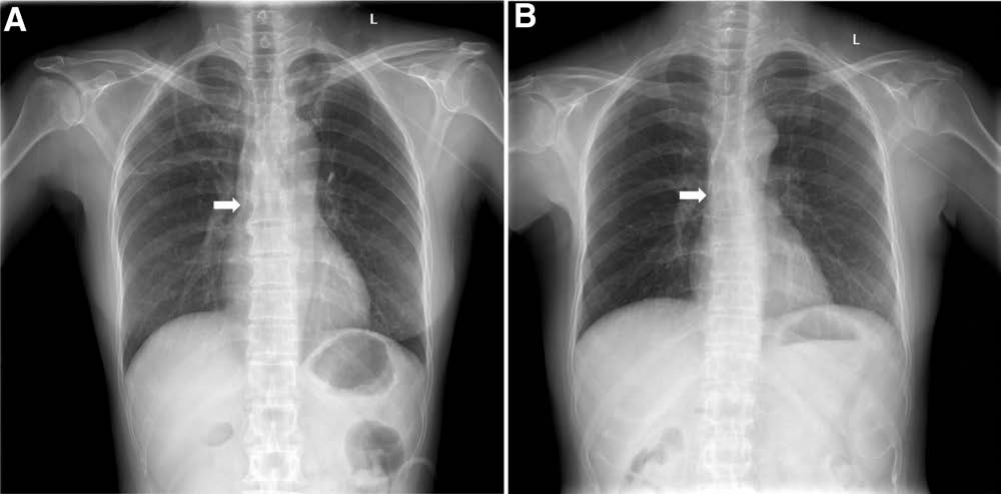
After the insertion of the PICC, Chest X-ray showed that the tip of the PICC was located at the lower third of the SVC (white arrows point to the tip of the PICC). (A) for patient A and (B) for patient B. PICC = peripherally inserted central catheter, SVC = superior vena cava.

A 50-year-old female with low differentiated adenocarcinoma of the pancreas (patient A) and a 51-year-old female with cystic neoplasms of the pancreas (patient B) were admitted to our hospital. The day before the operation, a 4-French power-injectable PICC (C.R. Bard, Inc., Murray Hill, NJ) was inserted with the ultrasound-guided modified Seldinger technique by an experienced PICC nurse, preparing for postoperative fasting and parenteral nutrition support. First, the diameters of the veins were evaluated by ultrasound. The left basilic veins of these 2 patients were chosen for insertion of the PICC since the brachial and basilic veins of the right upper arm had diameters <3.0 mm, which indicated a high risk of thrombosis. The insertion process was smooth, and an intracavitary ECG was used to track the tip of the PICC and confirm its correct position, with the amplitude of the P wave increasing to 60% to 80% of the QRS wave. For patient A, the catheter was 44 cm long, while for patient B it was 43 cm. Results of chest X-ray showed that the catheter tips were located at the lower third of the SVC (Fig. 1A and 1B).

**Figure 2.**
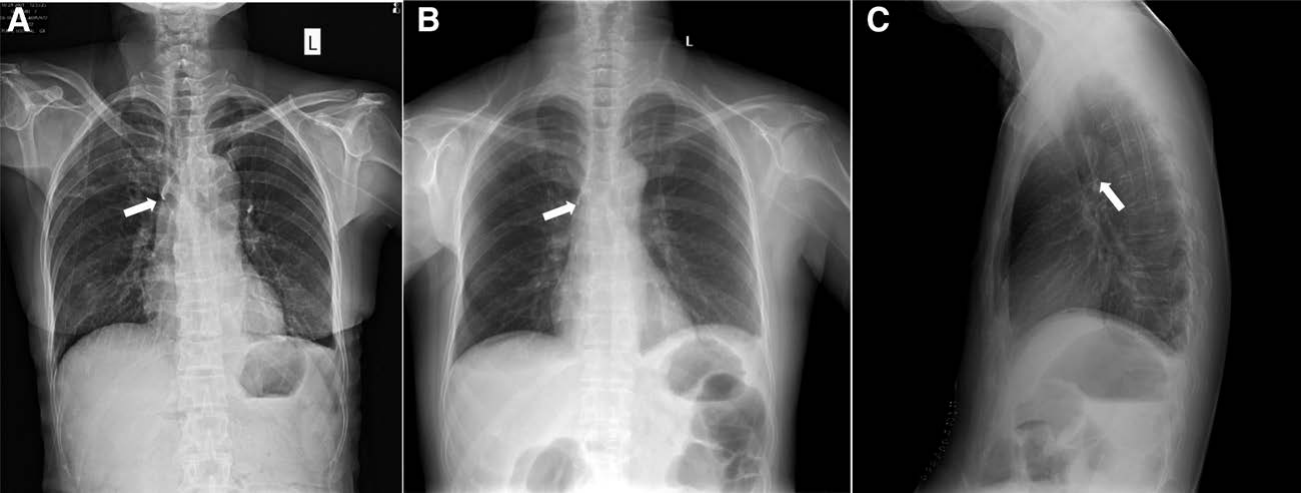
(A, B) Posterior/anterior chest X-ray films showed that the catheters of 2 patients were folded back (white arrows). (A) for patient A, (B) for patient B, and (C) The lateral chest X-ray of patient B clearly showed that the catheter was folded back and entered the azygos vein.

**Figure 3.**
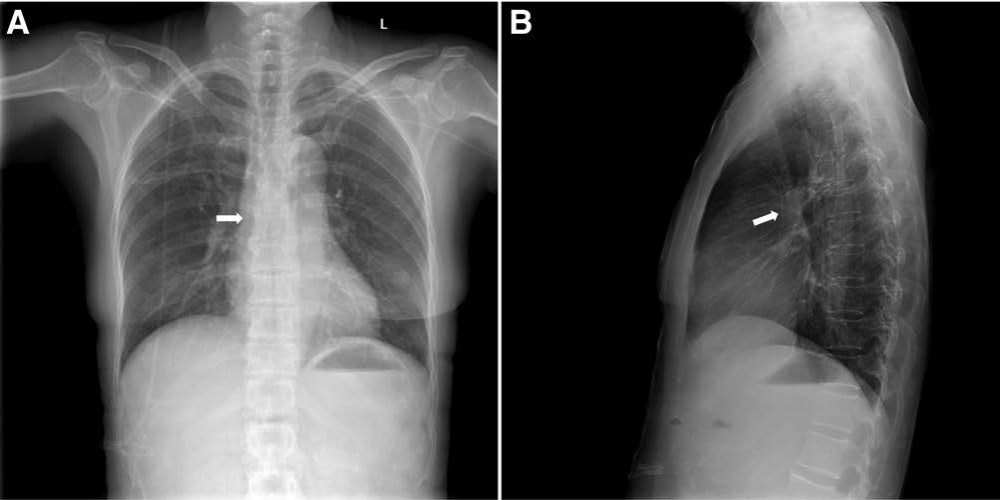
After the intervention, posterior/anterior and lateral chest X-rays of patient A confirmed that the tip of the PICC was restored to the lower third of the SVC (white arrows point to the tip of the PICC). PICC = peripherally inserted central catheter, SVC = superior vena cava.

Patient A underwent an open Whipple surgery and patient B underwent laparoscopic distal pancreatectomy and splenectomy. After surgery, patient A and patient B suffered from gastroparesis and constipation, respectively. Both patients denied experiencing nausea or vomiting after the implantation of PICC, and neither had gastroparesis or constipation prior to surgery. On the 8th day after patient A’s surgery, and the 6th day after patient B’s surgery, the nurses found that blood could not be aspirated from the PICCs although normal saline could be smoothly injected through the PICCs, and both patients had no complaints of discomfort during the injection. The transparent dressings on the catheter were intact and the exposed lengths of the catheter were the same as that when inserted. The circumferences of the left mid-upper arms were the same as before. Ultrasound showed that no “splash” was observed in the internal jugular vein after injection of normal saline, demonstrating no catheter malposition into the internal jugular vein. Subsequently, we used ultrasound to detect the route of the PICC in the left axillary vein and the left subclavian vein, showing no abnormalities. After that, both patients underwent intracavitary ECGs. The amplitude of the P wave exhibited no elevation, indicating that the PICC tip was not in the SVC. For both patients, we immediately took PA chest X-ray film and found that the catheters were folded back and the tips of the PICC were displaced into the azygos vein (Fig. 2A–C). Then the patients were in the semi-reclining position, and normal saline was injected into the PICC via a syringe (10 mL) with the push-pause flush technique, without any other adjustment of the catheter. Blood can be easily aspirated from the PICC after injection of 50 to 60 mL of normal saline. ECG was used again for confirmation of the location of the tip and presented with the elevated P wave of PICC, which was the same as that when inserted, indicating that the PICC tip reentered the SVC, located at the lower 1/3 of the SVC. PA and lateral chest X-rays were taken for patients, confirming the appropriate position of the PICC tip (Fig. 3A and 3B). Finally, the PICCs of the 2 patients functioned well afterward and were removed after the parenteral nutrition support was completed.

## 3. Discussion

The spontaneous PICC migration into the azygos vein is a rare PICC-related complication. As a branch of the SVC, the azygos vein originates from the right ascending lumbar vein and ascends along the right side of the thoracic vertebra, bending slightly forward at the level of the 4th or 5th thoracic vertebra, and drains into the SVC at its posterior wall. Before connecting the right brachiocephalic vein and forming the SVC, the left brachiocephalic vein flows from anterior to posterior. Therefore, the PICC inserted from the left limb goes through the left brachiocephalic vein and is more possible to enter the azygos vein compared to that from the right limb due to the smaller angle between the left brachiocephalic vein and the opening of the azygos vein.^[6]^ Anatomical factor may be a crucial prerequisite for spontaneous migration into the azygos vein because, in both of the cases we described here, the PICCs were placed from the left upper limb.

Coughing and severe vomiting have been reported to be related to spontaneous PICC migration, indicating that increased intrathoracic or intra-abdominal pressure might increase the risk of spontaneous PICC migration.^[3]^ However, the patients in this report did not suffer from coughing or vomiting, but 1 of them had gastroparesis and the other had constipation after surgery, indicating that they had postoperative gastrointestinal dysmotility. Approximately 30% to 40% of patients who undergo Whipple surgery have been reported to suffer delayed gastric emptying,^[7]^ and increased intraabdominal pressure may be a cause as well as a result of delayed gastric emptying. In laparoscopic surgery, gastrointestinal dysmotility is also associated with increased intra-abdominal pressure.^[8]^ Therefore, for patients who undergo abdominal surgery and have PICCs inserted in the left upper limb, the possibility of spontaneous migration of the PICC tip into the azygos vein should be considered when they have symptoms of gastrointestinal dysmotility after surgery and PICC dysfunction.

Evaluation of the function of PICC before catheter use is the basis for safety of PICC use, and blood withdrawal from the PICC is the primary method. Failure in blood withdrawals may suggest migration or fold-back of the PICC tip, and further analysis is necessary. When the migration of the PICC tip is suspected, intracavitary ECG can help find whether the tip is still in the lower part of the SVC, and ultrasound can facilitate to detect the PICC route and confirm whether the tip migrated in the internal jugular vein, which is the most common case. After that, a chest X-ray should be taken to confirm the position of the PICC tip. For migration into the azygos vein, the semi-reclining position can make the catheter tip move cephalad with a shorter part left in the azygos vein compared to the supine position.^[9]^ The push-pause flush technique can create turbulence within the catheter^[10]^ and pressure may result in the catheter tip out of the azygos vein and restore to its original position. However, for those patients who cannot cooperate, it may be inevitable to use the chest X-ray film to confirm the position of the PICC tip and withdraw the catheter tip from the azygos vein.

## 4. Conclusion

It is critical to evaluate the function of the PICC before every infusion. If a patient undergoes abdominal surgery with a PICC on the left side and suffers from symptoms of high intraabdominal pressure, such as gastrointestinal dysmotility, as well as the inability of blood withdrawals through the PICC, the possibility of spontaneous migration of the tip of the PICC into the azygos vein should be considered. Changing the posture of patients from the supine position to the semi-reclining position (or upright position, if possible), followed by flushing the PICC with the push-pause flush technique may help to restore the catheter tip into the SVC.

## Author contributions

**Conceptualization:** Weinan Liu, Wenyan Sun.

**Methodology:** Wei Wei, Yang Wang, Bing Liu, Wenyan Sun.

**Validation:** Weinan Liu, Yan Pang.

**Writing – original draft:** Wei Wei.

**Writing – review & editing:** Wenyan Sun.
